# An Ultra-Wideband Handover System for GPS-Free Bridge Inspection Using Drones

**DOI:** 10.3390/s25061923

**Published:** 2025-03-19

**Authors:** Ping-Hsiang Wang, Ruey-Beei Wu

**Affiliations:** 1 Graduate Institute of Communication Engineering, National Taiwan University, Taipei 106, Taiwan; r11942107@ntu.edu.tw; 2 Department of Electrical Engineering, National Taiwan University, Taipei 106, Taiwan

**Keywords:** ultra-wideband (UWB), handover system, UAV, bipartite graph, greedy algorithm, vertex coloring, internet of things

## Abstract

This study proposes an ultra-wideband (UWB) handover system that increases the range of UWB positioning for bridge inspection using an unmanned aerial vehicle (UAV). A bipartite graph and a greedy algorithm are used, and the problem is transformed into vertex coloring to address the challenge of a large number of anchors and insufficient anchor IDs because the area is long and there are numerous beams and columns under the bridge. Simulation and experiment show that the solution reduces the number of anchors that are required from 27 to 14, which significantly saves deployment costs and reduces power consumption.

## 1. Introduction

Bridge collapses and life-threatening accidents often occur because maintenance is neglected or there are natural disasters. Regular inspections can prevent these tragedies [1]. Initially, manual visual inspections were used, but this method is dangerous, features limited viewing angles, and struggles to accurately identify damage, especially in hard-to-reach areas [2].

To address these limitations, UAV technology is used for bridge inspections. Cameras capture images from beneath the bridges to detect cracks, dislocation, exposed steel, or concrete spalling. However, UAV operations under bridge usually face poor GNSS signal reception, which affects flight stability and the accuracy of image data [3].

Lidar is used for UAV localization but requires prior environmental mapping or computationally demanding simultaneous localization and mapping (SLAM). It has high horizontal resolution, but its effectiveness is reduced if the vertical height changes [4,5]. Image recognition technology has also been proposed for UAV positioning, but it requires significant computing resources, is affected by lighting conditions, and is inefficient if the field of view is obscured [6].

This study proposes the use of ultra-wideband (UWB) ranging technology for positioning under bridge where there is no GPS. This improves accuracy due to its high-frequency signal characteristics. UWB anchors are placed around the bridge and UWB tags are mounted on the UAV. Using trilateration, the UAV location can be calculated from the real-time distance between the tag and anchors [7].

UWB has high accuracy, but limited reception range. FCC regulations specify the maximum power of UWB chips as 41.3 dBm/MHz, which limits the transmission range to about 20 m without using a power amplifier [8]. On the other hand, it is easily susceptible to non-line-of-sight (NLOS) interference caused by obstruction, leading to ranging errors.

To reduce or avoid positioning errors of the DUT or minimize the number of anchors in NLoS situations, various anchor deployment algorithms can be employed [9,10,11,12]. A particle swarm optimization algorithm was used to enhance positioning accuracy and stability by optimizing anchor placement based on coverage and geometric dilution of precision [9]. A multi-objective deep Q-learning-based energy-optimized LoS/NLoS UWB node selection framework was proposed by efficiently handling the dynamic nature of indoor environments [10]. In addition, by considering the NLoS wave propagation model and employing a genetic algorithm, anchor locations were optimized to minimize the average root-mean-squares error in positioning [11]. Furthermore, a block coordinate-wise minimization algorithm was developed to minimize the number of anchors required to meet positioning accuracy in NLoS environments [12]. These methods are suitable for short ranges and thus basically consider indoor single-room and simple laboratory scenarios.

To increase the range of UWB for wider areas, such as bridge inspection routines, the RSSI value between the DUT and the master anchor is used to switch acting anchors [13]. However, the settings of master and slave anchors are limited, so more anchors are required, which reduces efficiency. Recently, a comprehensive solution combining UWB technology, cascaded wireless clock synchronization algorithm, and the positioning boundary optimization method for multiroom scenarios was proposed [14]. Another approach took advantage of the fusion localization by combining the inertial measurement unit (IMU) with UWB technology for LoS and NLoS detection and mitigation [15]. These methods basically distinguish LoS/NLoS by the received signal strength which is not decisive due to complex electromagnetic wave propagation.

This study proposes a UWB handover mechanism that exhibits efficient anchor placement, zone division, anchor grouping, and limited anchor encoding. It requires fewer anchors to ensure that the DUT receives UWB signals from sufficient anchors for all of its path. Also, many UWB modules have a limit on the maximum number of anchor IDs, so if the number of anchors exceeds the available IDs, it will not work. This method can be applied to a large area but will not cause interference due to duplicate anchor IDs. Therefore, the anchors with which the UWB tag is interacting can be clearly identified, effectively ensuring positioning accuracy.

The remainder of this study is organized as follows. Section 2 describes the architecture of the proposed UWB handover system. The positioning data for the device under test (DUT) is used to transition seamlessly between zones. Section 3 describes the anchor deployment and grouping algorithms that use a bipartite graph. To resolve ID conflicts, a vertex coloring problem (VCP) is constructed to group anchors in different zones. Section 4 gives simulation and experimental validation, followed by brief conclusions in Section 5.

## 2. Architecture of the UWB Handover System

### 2.1. UWB Handover Mechanism

**Figure 1:**
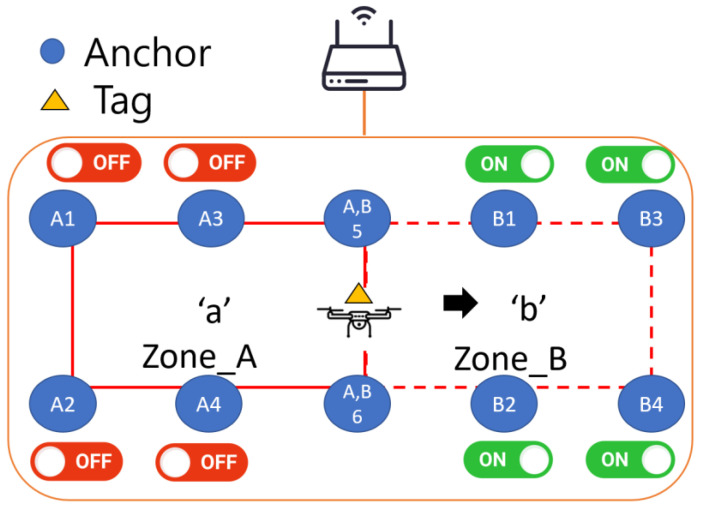
Example of handover mechanism. The host Rpi on UAV sends the character ‘b’ to all anchors when it moves from Zone_A to Zone_B. The Rpi on the anchor with corresponding character turns on the UWB module, otherwise it is turned off.

A handover mechanism groups the deployed anchors. The measured distance between the DUT and the anchors is used to obtain the position of the DUT, from which the zone where the DUT is located is obtained. The Raspberry Pi (Rpi) on the UAV transmits the broadcast character to the Rpi on all anchors in the zone. This part of the content is defined in advance, as shown in Figure 1.

The UWB module for this study is a GA210, which is a development version manufactured by GIPS Corporation. When the host Rpi on UAV receives the distance measurement from anchors, it calculates its position to determine the zone it belongs to, and then broadcasts a corresponding character to all anchors. If the Rpi on the anchor receives the characters that correspond to the zone where it is located, the control relay starts the UWB module; otherwise, it is turned off, as shown in Table 1. Specifically, if the UAV is in zone A, it transmits the character ‘a’ to all anchors. Only the anchors in zone A will be opened, while other anchors will remain closed.

Anchor names have two parts—English letters and numbers—as shown in Figure 1. The English letter indicates the zone to which it belongs, and the numeric part indicates its anchor ID in that zone.

Anchors that are located at the intersections of several zones are identified using several English letters.

### 2.2. Architecture of the Handover System

**Figure 2:**
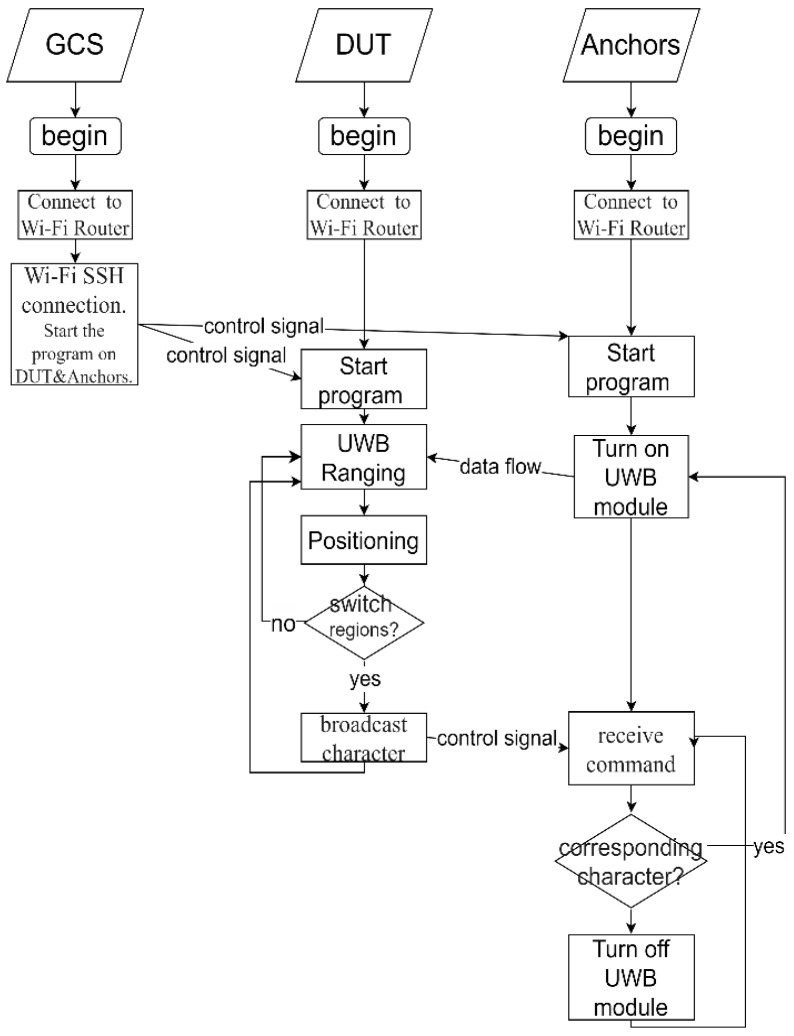
System control flowchart.

This subsection describes the control process for the anchors and the DUT in Figure 1. As shown in Figure 2, the ground station, DUT, and the anchors are connected to a Wi-Fi network. The ground station (GCS) issues instructions to execute the DUT and the local program of the anchor and then the anchors use the UWB module to perform ranging with the DUT to calculate the position of the DUT and determine whether to perform a handover. If there is a handover, the DUT issues instructions to the anchors to determine whether there is an instruction to turn on or off the UWB modules.

**Figure 3:**
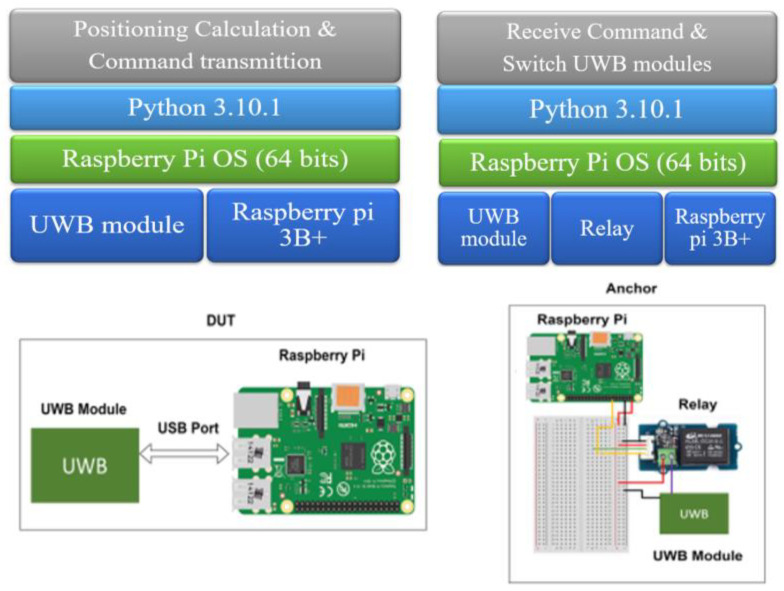
Software stack in DUT and anchors.

As shown in Figure 3, the hardware layer of the DUT is the UWB module and the Rpi 3B+, which are used to implement the positioning solution and to transmit commands. In the anchor part, the hardware layer is the UWB module, the relay, and the Rpi 3B+, which receive instructions and switch the task for the UWB module.

**Figure 4:**
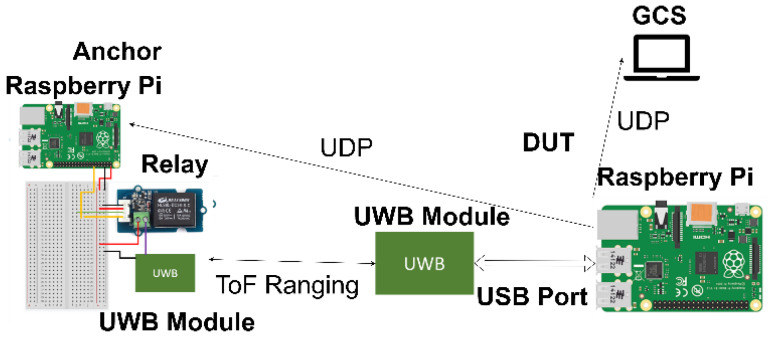
Diagram of the system architecture.

Figure 4 shows a system architecture diagram that details the communication protocol between various objects. The ground station controls the anchors and the DUT via the Secure Shell (SSH) [16] protocol to execute the program. The DUT issues instructions to the anchors via the UDP communication protocol to measure the range. The UWB module of the DUT in the DUT performs ToF ranging and the DUT transmits the positioning results back to the ground station.

## 3. Anchor Deployment and Grouping Algorithm

To use the UWB handover system to bridges, the anchors must be arranged and grouped. There are three main issues: (1) occlusion by the bridge which has many beams and columns and the effective range of UWB being than 40 m according to actual measurements; (2) deployment cost reduction by reducing the number of anchors that is deployed, even if the UWB signal covers the area under the bridge; and (3) the limited number of anchors that can be assigned at one time. The ID must be assigned to each anchor to avoid duplication of IDs in the same group, which causes ranging interference between the anchors.

### 3.1. Anchors Deployment in a Bipartite Graph Ubsection

The algorithm determines the location of the deployed anchors to allow multilateration. A bipartite graph ***G(C, S, E)*** whose vertices can be divided into two disjoint and independent sets, ***C*** and ***S***, is defined. The edges in the graph are represented by ***E***, each of which is connected to a vertex in the ***C*** set and a vertex in the ***S*** set. All locations where anchors are denoted as C-type vertices, and all possible activity points for the DUT are represented as S-type vertices.

Edges are then generated to construct the bipartite graph. For a UWB anchor at a candidate C-type vertex Cx, if the DUT at the S-type vertex Sx receives the UWB signal that is transmitted from Cx, an edge between is added between vertices Cx and Sx. This process is repeated for all possible C-type vertices, and the set of all added edges is the set ***E***.

For this study, positioning using multilateration employs 4 distances from the anchors to be measured. The value of 4 is selected to allow some redundancy to mitigate the measurement errors. The subset of ***C*** has the fewest anchors but is enough for all S-type vertices to find at least 4 edges. The solution is obtained using the greedy algorithm that was used in a previous study [17].

The RSSI value that is simulated using iBwave software ver. 4.1 is used in [17] to determine whether the anchor and the UAV are connected in the bipartite graph. The possibility that obstruction by obstacles in UWB ranging may cause multi-path interference is ignored. This study uses the distance between the UAV and anchors and the presence of any obstruction to determine the connection. The effective distance of UWB has been measured to be about 40 m, and the obstacles such as columns can be found from construction drawings and actual scenes.

### 3.2. Anchor Grouping by Vertex Coloring

**Figure 5:**
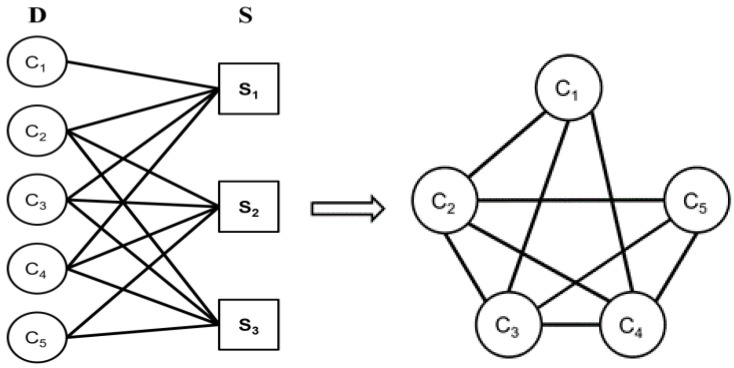
A toy example of transforming anchor grouping into a vertex coloring problem. The five C-type vertices C_1_ to C_5_ represent anchors, connected by three S-type vertices denoting possible UAV positions. Anchors connected to the same UAV position should be assigned different ID.

Figure 5 shows a toy example to demonstrate the transformation of anchor grouping into a vertex coloring problem. Specific C-type vertices are considered to be the vertices of the new graph. Using the bipartite graph relationship between these C-type vertices and zones, if two vertices are connected to the same S-type vertex, they are connected in the new graph; otherwise, they are not connected.

For the generated graph, colors are assigned from large to small, according to the number of connected vertices [18,19]. For the example in Figure 5, vertices C_1_, C_2_, C_3_, C_4_, and C_5_ respectively have 3, 4, 4, 4, 3 connections. Vertex C_2_ has the largest number of connections and is encoded as ID = 1. During the encoding, the ID code is not repeated if two vertices are connected, so vertices C_3_, C_4_, and C_1_ are sequentially encoded as ID = 2, 3, and 4. The last vertex C_5_ has a duplicate ID = 4 because it is not connected to C_1_.

After encoding, the region is divided into zones. For all the S-type vertices in the bipartite graph, if two S-type vertices are connected to C-type vertices of the same combination, the two are assigned into the same zone; otherwise, they are assigned to different zones. For example, vertex S_1_ in Figure 5 is connected to C_1_, C_2_, C_3_, and C_4_ and is named zone A, while vertices S_2_ and S_3_ are connected to the same combination of C_2_, C_3_, C_4_, and C_5_, and are named zone B. Finally, the anchors in Figure 5 are named as shown in Table 2. Vertices C_2_, C_3_, and C_4_ are all connect to S-type vertices in zones A and B and have English letters A and B in the names.

### 3.3. Algorithm Flowchart

First, obtain ***G(C, S, E)*** and set the parameters: minimum anchor number for each S-type vertex, x, and the weight θ. The algorithm determines the C-type vertices to be used as the anchors to form a set ***D***. The space state of an S-type vertex is also defined to represent the number of specified anchors to which it is already connected.

Initially, ***D*** is an empty set and the space states are all 0. The score of each C-type vertex is calculated by counting the number of S-type vertices to which it is connected. The vertex with highest score is added to ***D*** and the space state space for all S-type vertices is updated. This process is repeated until all space states are at least equal to x. The final bipartite graph ***G(D, S, E)*** is then constructed and it is determined whether C_i_ and C_j_ (i ≠ j) in ***D*** are connected to the same S-type vertex. If they are, C_i_ and C_j_ in ***G(D,E)*** are connected, and if not, the algorithm proceeds to the next step. Finally, obtain ***G(D,E)*** and solve it using the Vertex Coloring Problem (VCP).

The pseudo code is shown in Algorithm 1. The inputs are ***G(C,S,E)***, x, and *θ*. Lines 3 to 5 in the pseudo-code are initialization. For each C-type vertex, the initial fraction is *θ*(0) times the number of edges that are connected to it. The array *θ* is usually a decreasing array [17], such as *θ*(0) = 1, followed by the values 0.5, 0.25, 0.1, and 0 afterward.
**Algorithm 1:** Anchor Deployment and Grouping Algorithm.1 2 3 4 5 6 7 8 9 10 11 12 13 14 15 16 17 18 19 20 21 22 23 24 25 26 27 28 29 30 31 32Input: C, S, E, x, θ Output: [Anchor Position,Zone,Anchor ID] Set c.score in C to θ(0) * number of edges connected to c Set s.state in S to 0 D ← ∅, cover ← 0 while cover < S.size do c_chosen ← argmax (c.score for c ∈ C and c ∉ D)  for all s ∈ S adjacent to c_chosen do    s.state ← s.state + 1    if s.state = x then    cover ← cover + 1    for all c ∈ C adjacent to s and not in D do    c.score ← c.score + θ(s.state) − θ(s.state − 1)  Add c_chosen to D for all c in D do  for all d in D where c ≠ d do If c.adjacents ∩ d.adjacents ≠ ∅ then Connect c and d in the new graph G(V, E) for all c in G(V, E) do If c’s neighbors.ID have been assigned then c.ID ← the smallest integer ∉ c’s neighbors.ID else c.ID ← 1 AdjMap ←∅, ZoneMap ← ∅, curZoneID ← 1 for all s ∈ S do If s.adjacent ∈ keys in AdjMap then key ← values of s.adjacent in AdjMap ZoneMap[key] ← ZoneMap[key] ∪ s else AdjMap ← AdjMap ∪ {{s.adjacent}, curZoneID} ZoneMap ← ZoneMap ∪ {curZoneID, {s}} curZoneID ← curZoneID +

In line 6, the variable “cover” represents the number of S-type vertices that are connected to at least x vertices in ***D***. Lines 9 to 11 update the S-type vertex state and update the “cover”. Lines 12 to 13 update the score of the C-type vertex.

The variable “c.adjacent” in line 17 represents the set of all S-type vertices that are connected to c. Line 18 reviews the C-type vertices in ***D*** set to determine whether they are connected to the same S-type vertex. If they are, then in the newly generated graph ***G(V,E)***, two C-type vertices are connected, and vice versa. This will be used for the vertex coloring problem.

The variable “c.neighbor” in line 20 represents the set of all C-type vertices that are connected to c in ***G(V,E)***. Lines 21 to 23 assign a number to each C-type vertex. The variables “c.ID” represents the Anchor ID assigned to each C-type vertex. If c.neighbor has been assigned a number, the C-type vertex will be assigned the smallest integer greater than the assigned number.

The variables “AdjMap” and “ZoneMap” in line 24 are declared as two maps, each with a key and value. The variable “AdjMap” is used to map each C-type vertex combination to a specific area ID. The variable “ZoneMap” maps each specific area ID to all S-type vertices. Initially, both AdjMap and ZoneMap are empty. The variable “curZoneID” is used to represent different zones based on their values. Lines 25 to 32 divide the S-type vertices, where the variable s.adjacent represents the set of C-type vertices adjacent to s. If s.adjacent already exists in AdjMap’s keys, find the corresponding zone and assign the S-type vertex to that zone. If it does not exist, add s.adjacent to AdjMap, create a new zone, and associate that area with curZoneID. Then, assign the S-type vertex to this new area and increment curZoneID by 1.

## 4. Experimental Validation

The experiment has two parts. The first part designs an experiment to verify whether a handover system can be implemented. The second part shows the simulation results if the deployment and anchor grouping algorithm are applied in the area under the Wanshou Bridge in Taipei city.

### 4.1. Validation of the Handover System

**Figure 6:**
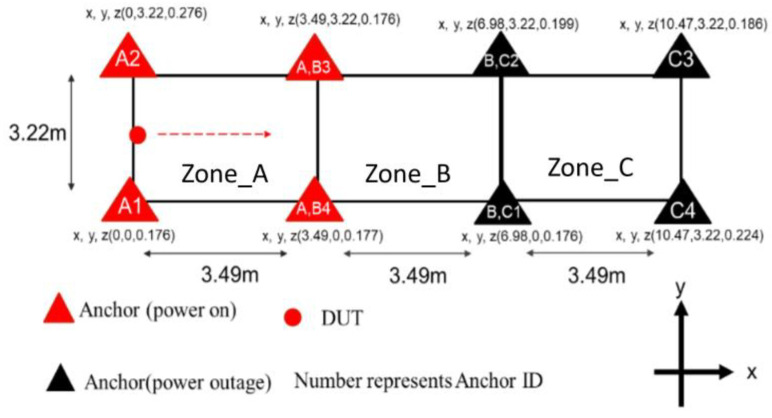
Schematic diagram of experimental setup. The DUT moves in the direction of the dashed arrow. While it is in Zone_A, anchors that have corresponding character A are turned on, while others off.

**Figure 7:**
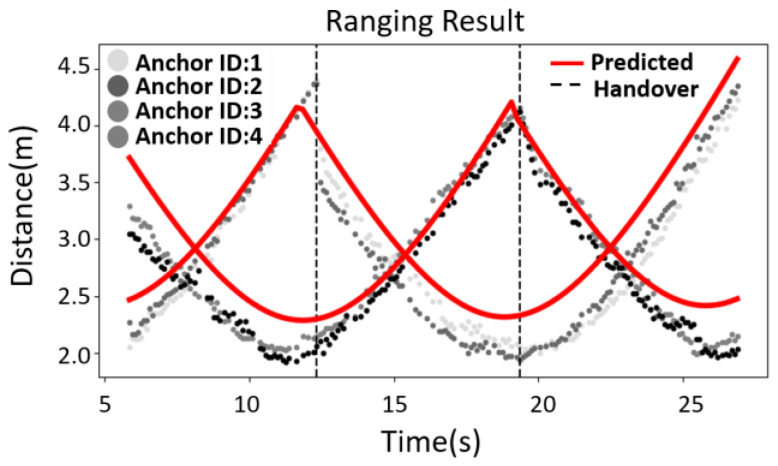
Experimental verification for the predicted ranging results in anchors with ID = 1, 2, 3, 4.

As shown in Figure 6, two sets of UWB anchors with IDs of 1, 2, 3, and 4 were deployed in the atrium of Mingda Hall 5F in National Taiwan University in an area of 10.47 m × 3.22 m. Using triangulation, each of the four anchors locate the UWB tag, so this experiment divides the region into three zones and a handover occurs at two boundaries. The handover mechanism uses the current positioning value for the DUT in the x-direction and is executed if the positioning value falls at x = 3.94 or x = 6.98. As shown in Figure 7, if the DUT is located within a certain zone, the UWB anchors in that zone are switched on, while the remaining UWB anchors are switched off.

For the experiment, a Rpi that is equipped with a UWB tag was carried in a straight line from the (x, y) coordinate (0, 1.61) to (10.47, 1.61). The handover mechanism was activated along the way. The ranging results and positioning calculation results from the anchors with ID = 1, 2, 3, and 4 were recorded for subsequent analysis.

The ranging results for the anchors with ID = 1, 2, 3, and 4 are shown in Figure 7. The horizontal axis shows time and the vertical axis shows the ranging result (in meters). The red line in the figure is the estimated ranging value, the black dots are the actual ranging result from the packet, and the dotted lines are the time points for the handover. There are positioning errors, so the first and second handover times are longer than expected.

The handover processes take 48 ms and 46 ms, respectively, which is less than 100 ms of the ranging period. The experimental results show that the DUT maintains continuous ranging with the anchors, and the trend for the ranging results is significantly different after the time point of handover. This demonstrates that the DUT converts ranging from the anchors in the original zone to the anchors in the next zone. The handover must be very fast to achieve continuous ranging, so the UDP communication protocol is used.

**Figure 8:**
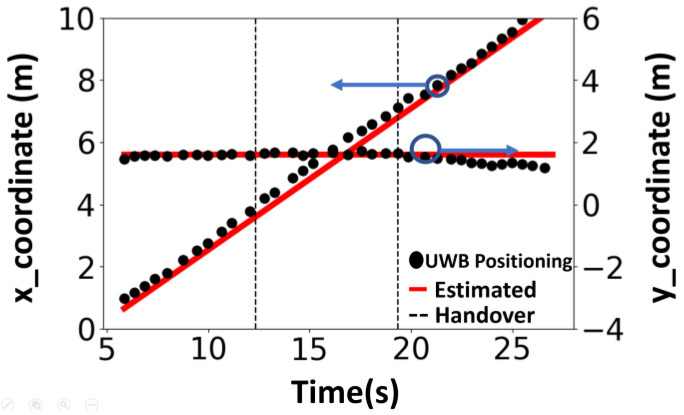
Diagram of the positioning solution result. The DUT moves along *x* direction. The estimated position (x, y) over time is plotted using UWB measurement with handover and compared to the estimated route (red lines).

Figure 8 shows the (x, y) coordinates for the positioning result. The horizontal axis shows time and the vertical axis shows the x coordinate and the y coordinate in units of meters. The red lines denote the x coordinate and y coordinate of the estimated location of the DUT, the black dots are the x and y coordinates of the actual positioning solution result, and the black dotted line marks the time point of handover. Positioning accuracy can be estimated by comparing the estimated position with the actual routes. The RMSE is 0.102 m for the x-directed route, and 0.176 m for the inclined route.

### 4.2. Simulation of the Deployment and Anchor Grouping Algorithm

**Figure 9:**
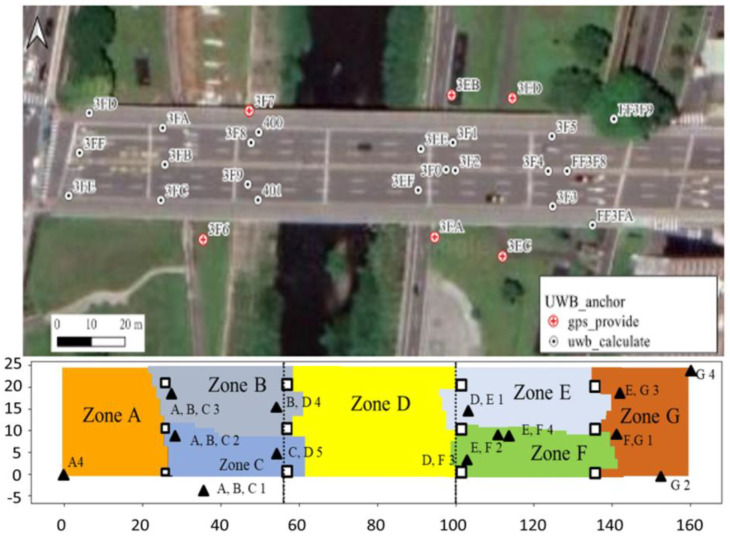
Anchor deployment and grouping before and after using handover algorithm for the Wanshou Bridge. Different colors denote different zones. The triangles denote the selected anchors, and the letters and numbers represent the name of activating zones and anchor ID.

This study uses anchor IDs so the anchor IDs are assigned to prevent ranging interference. As shown in Figure 9, before the algorithm is used, 27 anchors are deployed, and are shown as circles in the upper figure, to ensure that the UWB signal extends over the entire Wanshou Bridge.

To begin with, there are 100 possible anchor placement locations under the bridge through random sampling, which are treated as C-type vertices in the bipartite graph. The entire activity space under the bridge is divided into 4752 non-overlapping areas, and each 1.0 m × 1.0 m square area contains an S-type vertex. Edges are generated based on the distance between anchor locations and the S-type vertices and whether there are obstacles in between. The topology of the selected anchors is then produced by applying Deployment and Anchor Grouping Algorithm.

After the algorithm is executed, only 14 anchors are required, and these are shown as triangles in the lower figure. The anchor ID only uses five codes, which is fewer than the eight codes that are available in the model GA210 Anchor ID development version.

### 4.3. Indicators of Deployment Quality

Since the present method aims to minimize the number of IDs required, the resultant anchor positions are not optimal. Hence, the quality of deployment deserves further assessment. The first aspect involves the dispersion of the selected anchor locations. When the UAV is closer to the center of the anchor network, the localization error is smaller [20]. However, if nearby anchors are too close to each other, this may result in increased positioning errors. Therefore, we calculate the distance between each anchor and its two nearest anchors [17]. The results show that these distances range from 2.7 to 33.3 m, with a standard deviation of 8.3 m and an average of 18.1 m. Compared to the ideal value of 40 m, most anchors are sufficiently spread out, but a few anchors are too close, such as E,F2, and E,F4, which reduces the overall average distance. It may not be so critical since at least four anchors are selected for each zone. However, this indicates that the algorithm still has room for improvement.

**Figure 10:**
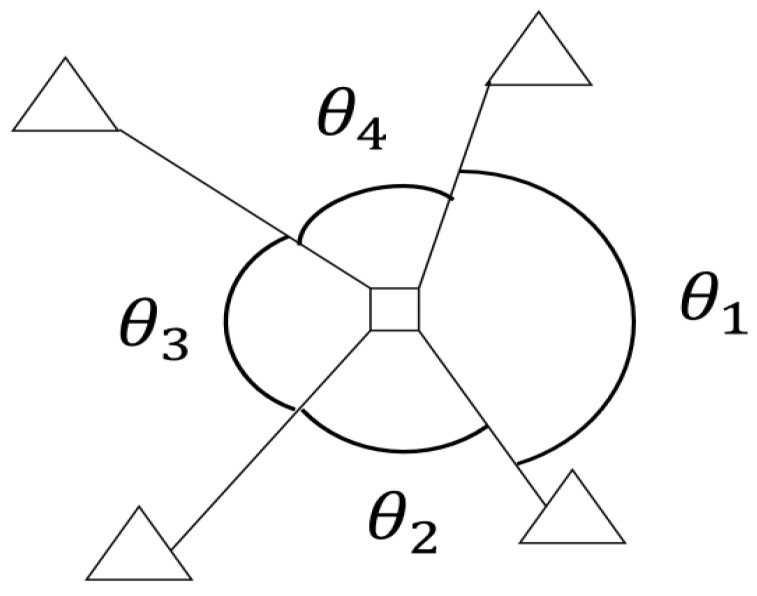
Definition for separation angle. The triangles represent anchors, and the square is the gravity center of four anchors in the same zone.

The second aspect is the relative angle of the anchor positions within each zone. For more accurate positioning, the UAV needs to receive anchor signals from different directions. We connect the gravity center of each zone to the UWB anchors whose signals can be received and define the “separation angle” as the maximum relative angle. The smaller the separation angle, the more dispersed the signals are. Taking Figure 10 as an example, the four triangles represent anchors, from which the signals can be received at the gravity center of the quadrilateral, and the separation angle is the maximum relative angle θ_1. The separation angle in Figure 9 ranges from 122.2° to 178.9°, with an average of 157.2°. Although the algorithm reduces the number of anchors, the placement is affected by obstacles and there is still room for improvement in achieving the ideal angle of 90°.

## 5. Conclusions and Discussion

This study proposes a UWB handover system that uses a UWB, relays, and Rpi 3B+ modules. Anchors are connected using a Wi-Fi network and a low-latency UDP protocol is used so the system achieves handover times of less than 0.05 s, which is faster than the UWB ranging cycle of 0.1 s. This allows seamless positioning of the DUT over different zones.

An anchor placement algorithm uses a bipartite graph, a greedy algorithm, and a vertex coloring problem to allow region partitioning and anchor selection. The simulation results show that this algorithm significantly reduces the number of anchors that are required and the associated costs.

It is worth noting the issues of limited positioning accuracy in complex environments because, despite reducing the number of anchors, the method may still encounter difficulties in precisely determining the UAV’s position in areas with numerous obstacles, such as beams and columns under the bridge, which can lead to UWB signal interference and localization errors. Additionally, UWB technology is susceptible to signal interference caused by reflections and issues related to NLoS. Furthermore, the approach described in the article relies on a fixed placement of anchors, which may prove ineffective in situations where conditions change dynamically, such as varying weather or the movement of objects under the bridge.

This study adopts a remedy to address these difficulties. Positioning using multilateration employs four distances from the anchors to be measured. Since theoretically only three anchors are needed to determine the position, the chosen value of four allows some redundancy to mitigate the measurement errors. If there should be one or more distance errors, it can be detected as an outlier [21] and will not be used to decide the change in zones. Nonetheless, further optimization of the algorithms or adaptive anchor placement strategies are still needed to enhance the system’s reliability and resilience in various challenging operational conditions. These deserve further study.

## Figures and Tables

**Table 1:** State transition of UWB anchors.

Received State	Power On	Power Off
**corresponding character**	Power on	Power on
**non-corresponding character**	Power off	Power off

**Table 2:** Anchors grouping via vertex coloring.

Anchor	C_1_	C_2_	C_3_	C_4_	C_5_
name	A4	A,B1	A,B2	A,B3	B4

## Data Availability

Data are contained within the article.
